# Investigating Behaviour and Population Dynamics of Striped Marlin (*Kajikia audax*) from the Southwest Pacific Ocean with Satellite Tags

**DOI:** 10.1371/journal.pone.0021087

**Published:** 2011-06-14

**Authors:** Tim Sippel, John Holdsworth, Todd Dennis, John Montgomery

**Affiliations:** 1 School of Biological Sciences, University of Auckland, Auckland, New Zealand; 2 Blue Water Marine Research, Whangarei, New Zealand; University of Maryland, United States of America

## Abstract

Behaviour and distribution of striped marlin within the southwest Pacific Ocean were investigated using electronic tagging data collected from 2005–2008. A continuous-time correlated random-walk Kalman filter was used to integrate double-tagging data exhibiting variable error structures into movement trajectories composed of regular time-steps. This state-space trajectory integration approach improved longitude and latitude error distributions by 38.5 km and 22.2 km respectively. Using these trajectories as inputs, a behavioural classification model was developed to infer when, and where, ‘transiting’ and ‘area-restricted’ (ARB) pseudo-behavioural states occurred. ARB tended to occur at shallower depths (108±49 m) than did transiting behaviours (127±57 m). A 16 day post-release period of diminished ARB activity suggests that patterns of behaviour were affected by the capture and/or tagging events, implying that tagged animals may exhibit atypical behaviour upon release. The striped marlin in this study dove deeper and spent greater time at ≥200 m depth than those in the central and eastern Pacific Ocean. As marlin reached tropical latitudes (20–21°S) they consistently reversed directions, increased swimming speed and shifted to transiting behaviour. Reversals in the tropics also coincided with increases in swimming depth, including increased time ≥250 m. Our research provides enhanced understanding of the behavioural ecology of striped marlin. This has implications for the effectiveness of spatially explicit population models and we demonstrate the need to consider geographic variation when standardizing CPUE by depth, and provide data to inform natural and recreational fishing mortality parameters.

## Introduction

Striped marlin (*Kajikia audax*) [1] (Family: Istiophoridae) are important components of targeted (recreational and commercial) and non-targeted (bycatch) fishing activity throughout the Indo-Pacific. Their highly migratory nature means tagging is a critical component of understanding both their behavioural ecology and population biology. Two kinds of electronic tags, 1) archival, which collect high frequency environmental data (ambient temperature, depth, light-level); and 2) satellite-linked radio telemetry (SLRT), which provide high spatial resolution movement data, have become important tools for investigating the movement ecology of highly migratory pelagic species. Time-series models developed to approximate movements of tagged animals from archival tag data have provided important new biological insights, but both theoretical and practical limitations constrain the spatial resolution of this approach. Double-tagging enables the strengths of each technology to be utilized simultaneously [2], [3]. Recently developed methods for electronic double-tagging of billfish [4] can provide very good inputs for behavioural classification models, and with the high cost and effort invested in collecting marine telemetry data, methods for extracting as much information as possible from the data are needed. Research into techniques for classifying behaviours from telemetry data is increasing rapidly [5]–[8], and analytical approaches are evolving, but standardized methods for classifying behaviour are yet to emerge [9]. These methods may contribute valuable insights into factors affecting foraging and migration ecology, and provide opportunities to examine important aspects including post-release behavior, depth distribution, and linkages between individual movements and geographic distribution.

Satellite tagging has been used to characterize the geographic distribution and general movement patterns of adult striped marlin from the Pacific Ocean over 1–9 month periods [10], and acoustic tracking has shown movement and behaviour trends at local scales over brief periods (up to 52 hours) [11], [12]. However, research into relationships among the behaviour of individuals, spatial distributions of the population, and environmental conditions over longer periods can greatly improve biological and ecological understanding, as well as management of the species.

Electronic tags have been used widely to investigate the survivorship of billfish following release from recreational [13]–[15] and commercial fishing gear [16]–[18]. However, very little information is available about potential capture and tagging effects on the behaviour of wild marine animals, despite the potentially important consequences for interpretation of telemetry studies, and management measures derived from them. The assumption implicit in most animal telemetry research is that the behaviours of animals that survive capture and tagging are representative of their broader untagged populations. The degree to which this assumption is valid may vary among species and tagging methods, but clearly has implications for interpretation of data derived from electronic tagging.

Information about regional variation of striped marlin occupancy of water column can significantly impact standardization of catch-per-unit-effort (CPUE) data and efforts to mitigate bycatch. Although known to spend the majority of their time near the surface, less is known about their utilization of other water column strata. Utilization of the water column by pelagic fishes tends to be shallower in the eastern tropical Pacific (ETP) than in most of the rest of the Pacific because of a sharp vertical oxycline and shallow thermocline [19]. As a result, maximum depths of striped marlin in the ETP are shallower [14] than the southwest Pacific [20]. Additional tagging can add to these datasets and help define water column use by striped marlin.

The geographic distribution of Pacific striped marlin has been inferred from commercial longline CPUE and conventional tagging data [21], [22]. Characterized as ‘horse-shoe shaped’, it is continuous across the equator in ETP, but becomes meridionally discontinuous from the central Pacific and westward. However, insufficient spatial and/or temporal resolution in tagging data [11], [12], [23] have precluded the ability to link individual movements and behaviour with this distinct pattern.

Double-tagging data are used here to investigate the movement patterns of striped marlin within the southwest Pacific Ocean. A model is developed to classify pseudo-behavioural states and then to infer the frequency and duration of area-restricted and transiting behaviours. As a result, a basis for investigating their response to the capture/tagging process is also established. We show how different behavioural modes are related to swimming depths. Relationships among the behaviour of individuals and the distribution of the population are illustrated for the first time. This study provides the detailed analysis of long-term behaviour reported thus far, describing new insights into the biology and ecology of striped marlin. We discuss how this research can inform population dynamics models including spatio-temporal stratification, depth standardized CPUE, recreational fishing mortality, and natural mortality.

## Materials and Methods

### Animal ethics

This research was approved under permit AEC-R431, issued by the Animal Ethics Committee of University of Auckland School of Biological Sciences.

### Capture and tagging

Striped marlin were caught between 2005–2008 from recreational fishing vessels as described in Holdsworth *et al* [4]. Tagging was completed while fish remained in the water alongside the boat during 2005, 2007, 2008; in 2006 fish were brought aboard and onto a padded deck mat via a stern ramp. A combination of pop-off satellite archival tags (PSAT)s and SLRT were used to investigate their movements and behaviour patterns. PSATs were either model PAT3 (2005) or PAT4 (2006–2008), SLRT tags were model SPOT5, and all were manufactured by Wildlife Computers (Redmond, Washington, U.S.A.). PSATs weighed 75 g in air and were 750 mm long (excluding antenna) with a maximum diameter around the float of 40 mm, while SPOT tags weighed 32 g in air with the dimensions 80×19.5×10.5 mm. PSAT tags were tethered by 300 lb monofilament fishing line to plastic (vinyl) intra-muscular anchors which were implanted between dorsal pterygiophores of the fish, with a secondary anchoring point created by a looped conventional tag at the base of the float to hold the PSAT closer to the fish body. PSATs recorded water temperature, depth and sunlight intensity every 30–60 seconds and were programmed to summarize their data into 12 summary temperature and depth bins, divided into 3, 6, or 12 hour intervals at transmission. SPOT tags were mounted inside vinyl sleeves which were slipped over the upper tail lobe and stapled or bolted on [4].

### Data analysis

All data analyses were conducted using the statistical environment **R** [24]. Use of functions from external packages are cited as ‘function(**package**)’. Unless stated otherwise, averages are noted as mean ± standard deviation.

### Location estimates

SLRT locations were computed directly by Argos satellites and properties of these positions (indices of quality and associated error structures) have been assessed extensively [6], [25]. Light-based geo-positions from PSATs were approximated using the tag manufacturer's proprietary software WC-GPE, which implements threshold light-level geolocation methods [26], [27]. When estimating light-based geolocations from double-tagged animals, Argos quality mid-point positions from SLRT tags were used to calibrate these estimates in WC-GPE. The initial positions and sea-surface temperatures (SST) recorded simultaneously by the PSATs were used as inputs into an SST cross-referenced Kalman filter in the package **uKFSST** [28], [29]. The magnitude and distribution of errors from Kalman filter location estimates for striped marlin has been assessed previously [4].

### Temporal regularization of trajectories

Most telemetry studies record observations at irregular intervals, and it is common to use inferential models which require regular time-steps to analyze latent pseudo-behaviours [6], [30], [31]. We also took this approach. A regular time-series of locations was estimated from irregular observations for each animal using a continuous-time correlated random walk Kalman filter (CTCRW hereafter) from the package **crawl** [32], [33]. From the continuous time-series, locations were extracted at 12-h intervals (00:00 and 12:00) over the course of the entire animal track, or similar to the average 2.3 locs/day of these data [4]. Appendix S1 includes further detail about regularization.

### Classification of behaviour modes

A movement model was formulated to infer four discrete behavioural states; “slow-transiting“, “fast-transiting“, “slow-ARB“, “fast-ARB“ (ARB is short for area restricted behaviour). Trajectory segments with high turning angles have been referred to as ‘area-restricted search’ (ARS) previously [34], [35]. The term ‘search’ is suggestive of a specific kind of behavior (searching), but the animal may or may not have found what it ‘searches’ for at any moment and classifying resting as a form of ‘search’ seems undesirable. As a result, the term ‘area-restricted behavior’ (ARB) is used as a more generic reference to area-restricted patterns. Appendix S1 details further the procedure of classifying behaviours.

### Statistical analysis

Comparisons were made among the behaviours inferred by the model and distributions of maximum depths reported by PSAT tags. Profiles of depth and temperature (PDT)s, were transmitted by PSAT tags at 8 discrete depths (and mean temperature at depth) distributed between the minimum and maximum during pre-programmed summary periods. Maximum depths were chosen for analysis because they are the most commonly observed discrete measure of position in the water column across all animals. Maximum depths were non-normally distributed, so non-parametric tests for asymptotic distributions (analogous to t-tests for normally distributed data) were used to compare maximum depths with transiting and ARB behaviours modes (without respect to speed). Probability-density plots of time spent within discrete depth bins summarized over the same temporal periods described above were used to illustrate changes in depth distributions over time. Gaps in the depth data bins were interpolated using the function loess(**fields**) [36].

The probability of occurrence of ARB with respect to the number of days at liberty was estimated by fitting a proportional hazards model to produce a Kaplan-Meier survival curve using the function Surv(**survival**) [37]. Behaviour was coded as a binary variable (0 = not ARB, 1 = ARB) using days at liberty (or days since release, DAL hereafter) as the time variable and ordinal date as the predictor.

## Results

Twenty-eight striped marlin were tagged, but three were excluded from analysis due to poor data quality or post-release mortality. Twenty-five datasets collected during the austral summers of 2005 (*n* = 3), 2006 (*n* = 15), 2007 (*n* = 5), and 2008 (*n* = 2) were included in the analysis (Table 1). Fifteen datasets were collected from individuals fitted with PSAT and SLRT tags simultaneously, and 10 SLRT only datasets were obtained. SLRT tracks averaged 32 days in length (range 1–114 days) and a mean of 25 PSAT geolocation days (range 0–133 days at liberty) per fish was also recorded. DAL ranged from 15 to 133 days, totaling 1432. A total of 1042 locations from SLRT and PSAT tags were observed on 398 different days.

**Table 1:** Striped marlin included in behavioural analysis.

Marlin	Weight	Start	Start	Start	End	End	Days At	SLRT	PSAT	Max Gap
ID	(kg)	Date	Lat.	Lon.	Lat.	Lon.	Liberty	Days	Days	Days
STM05.2	85	25 Feb 05	−37.244	176.103	−31.245	173.185	21	9	21	11.0
STM05.3	100	26 Feb 05	−37.295	176.273	−29.506	180.460	21	21	21	2.5
STM05.4	70	18 Mar 05	−37.069	176.039	−21.703	176.134	60	25	60	2.0
STM06.1*	74	10 Jan 06	−32.635	167.563	−31.897	167.068	68	25	68	1.0
STM06.2	80	11 Jan 06	−31.683	167.833	−31.368	168.824	15	1	15	4.1
STM06.3	81	12 Jan 06	−31.707	167.835	−33.013	167.582	103	21	103	11.0
STM06.4**	66	13 Jan 06	−31.744	167.880	−33.443	180.484	28	28	0	4.1
STM06.5	110	4 Feb 06	−36.513	173.629	−24.105	167.376	80	21	80	41.0
STM06.6	66	20 Feb 06	−37.646	177.813	−24.191	177.850	65	23	65	2.3
STM06.7	66	1 Mar 06	−37.397	176.374	−24.552	182.792	85	43	85	19.0
STM06.8**	65	31 Mar 06	−31.706	167.846	−20.217	168.878	28	28	0	14.1
STM06.9**	81	13 Feb 06	−34.858	173.757	−32.638	173.053	17	17	0	6.4
STM06.10	75	31 Mar 06	−31.706	167.846	−21.717	161.812	133	36	133	7.0
STM06.11	95	31 Mar 06	−31.707	167.852	−23.757	175.176	68	25	68	12.0
STM06.12*	80	2 Apr 06	−31.709	167.833	−26.22	170.119	34	17	34	2.0
STM06.13	90	3 Apr 06	−31.667	167.817	−26.275	172.453	29	15	29	2.0
STM06.14	80	3 Apr 06	−31.683	167.850	−21.817	170.404	100	26	100	3.0
STM06.15**	76	4 Apr 06	−31.699	167.845	−27.179	166.796	18	18	0	2.3
STM07.1***	85	19 Feb 07	−37.485	177.982	−20.111	225.358	103	103	0	6.7
STM07.2***	60	19 Feb 07	−37.408	178.344	−30.682	182.985	59	59	0	19.8
STM07.3***	75	20 Feb 07	−37.509	177.847	−25.628	169.307	50	50	0	2.0
STM07.4	100	20 Feb 07	−37.560	177.660	−27.422	202.586	91	33	91	7.0
STM07.5***	80	21 Feb 07	−37.373	177.754	−34.448	180.691	18	18	0	1.6
STM08.1**	80	6 Mar 08	−37.449	177.964	−22.119	182.035	115	115	0	6.2
STM08.2**	75	8 Mar 08	−37.509	178.073	−27.701	179.716	22	22	0	1.5
Mean	79.8						57	32	39	8
Total							1431	799	973	

*Archival PSAT data were recovered.

**PSAT didn't transmit.

***Animal was single-tagged with SLRT only.

### Data quality

Temporal coverage of individual marlin was variable, with maximum duration for missing data from individual fish ranging from 1–41 days (7.6±8.8). The highest quality location data generally occurred within the first 4–8 weeks of the tracks when SLRT locations were available. Exceptions to this included STM06.8 and STM07.2, which had gaps of 14 and 20 days at the beginning of their tracks. STM07.1 and STM08.1 provided exceptionally good SLRT data for 103 and 115 days (max gaps 6.7 and 6.2 days) respectively (Table 1). Root mean squared (RMS) error for CTCRW smoothed uKFSST location estimates from transmitted PSAT data were 12.5 km and 87.8 km for longitude and latitude respectively. RMS errors for CTCRW smoothed uKFSST locations from archival PSAT data were 214.6 km and 30.0 km for longitude and latitude, respectively. The large overall RMS errors for archival longitudes was due to a bias over the initial 10 days at liberty for STM06.1 (longitude RMS = 264.6 km), but were much better and consistent with expectations for STM06.12 (longitude RMS = 31.8 km).

### Behaviour classification

**Figure 1:**
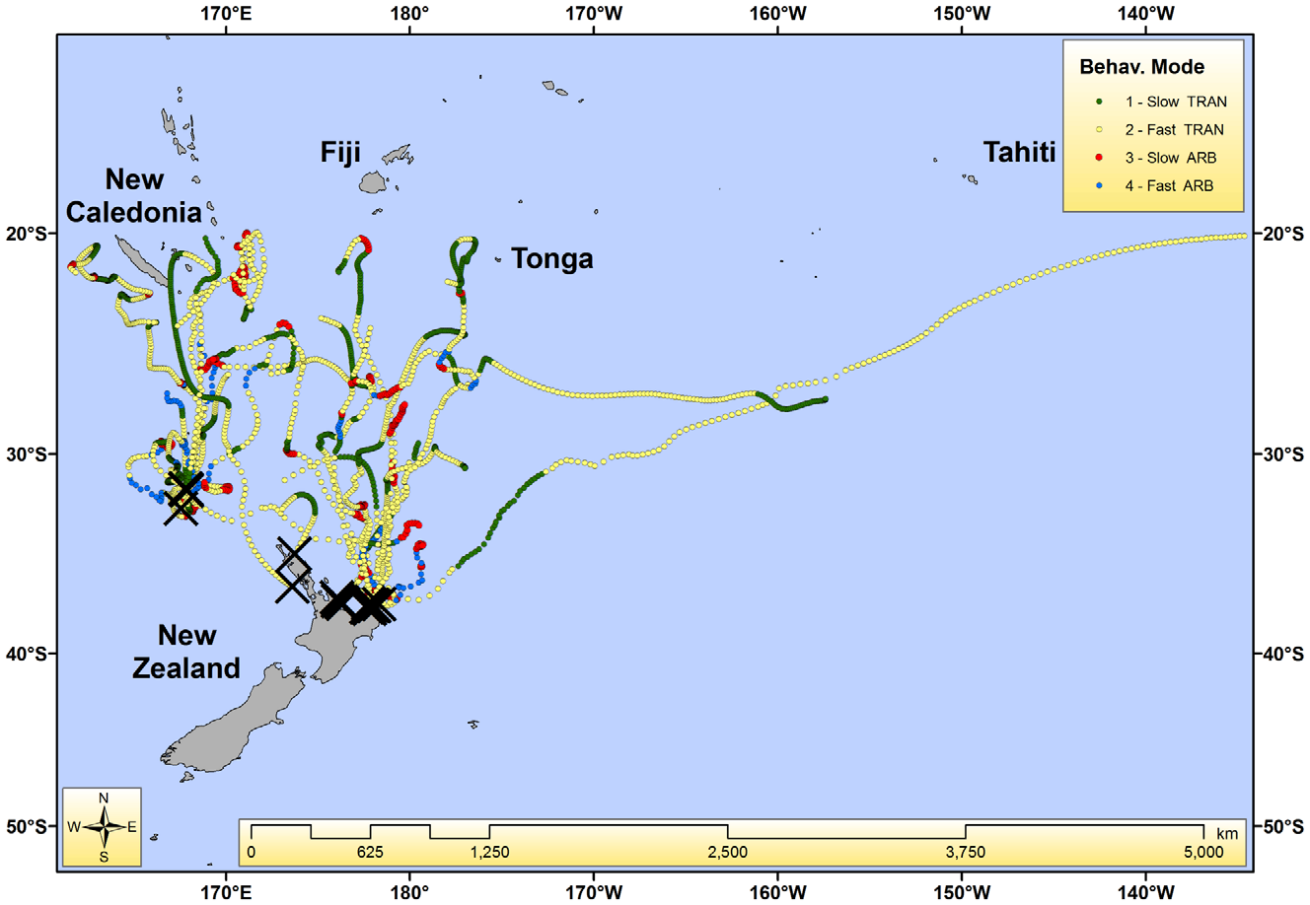
Twenty-five striped marlin trajectories colour coded by inferred modes of behaviour (X represents initial capture location).

All four behavioural modes were predicted, but not all were observed in each individual (Figure 1). The number of behavioural modes inferred per individual ranged from 2–4. Mean travel speeds for slow-transit and fast-transit modes were 0.74±0.56 km/hr (range 0.0–3.5 km/hr) and 1.93±1.04 km/hr (range 0.0–8.5 km/hr), respectively. Continuous durations of segments of ARB lasted from 1 to 19.5 days (5.5±4.8 days). The number of days between ARB events ranged from 1.5 to 50 (14.3±13.3). Fast-ARB (mode 4) is probably heavily influenced by location error, as traveling rapidly in a highly tortuous manner for prolonged periods is not biologically realistic. We do not consider fast-ARB to be a distinct behaviour, but rather a mode which is useful for identifying trajectory segments more heavily influenced by high location error.

### Representative tracks

Results for at least one marlin from each tagging season (2005–2008) are discussed. Two archival datasets were recovered from fish tagged in 2006 (Table 1), and analysis from one of these is included.

**Figure 2:**
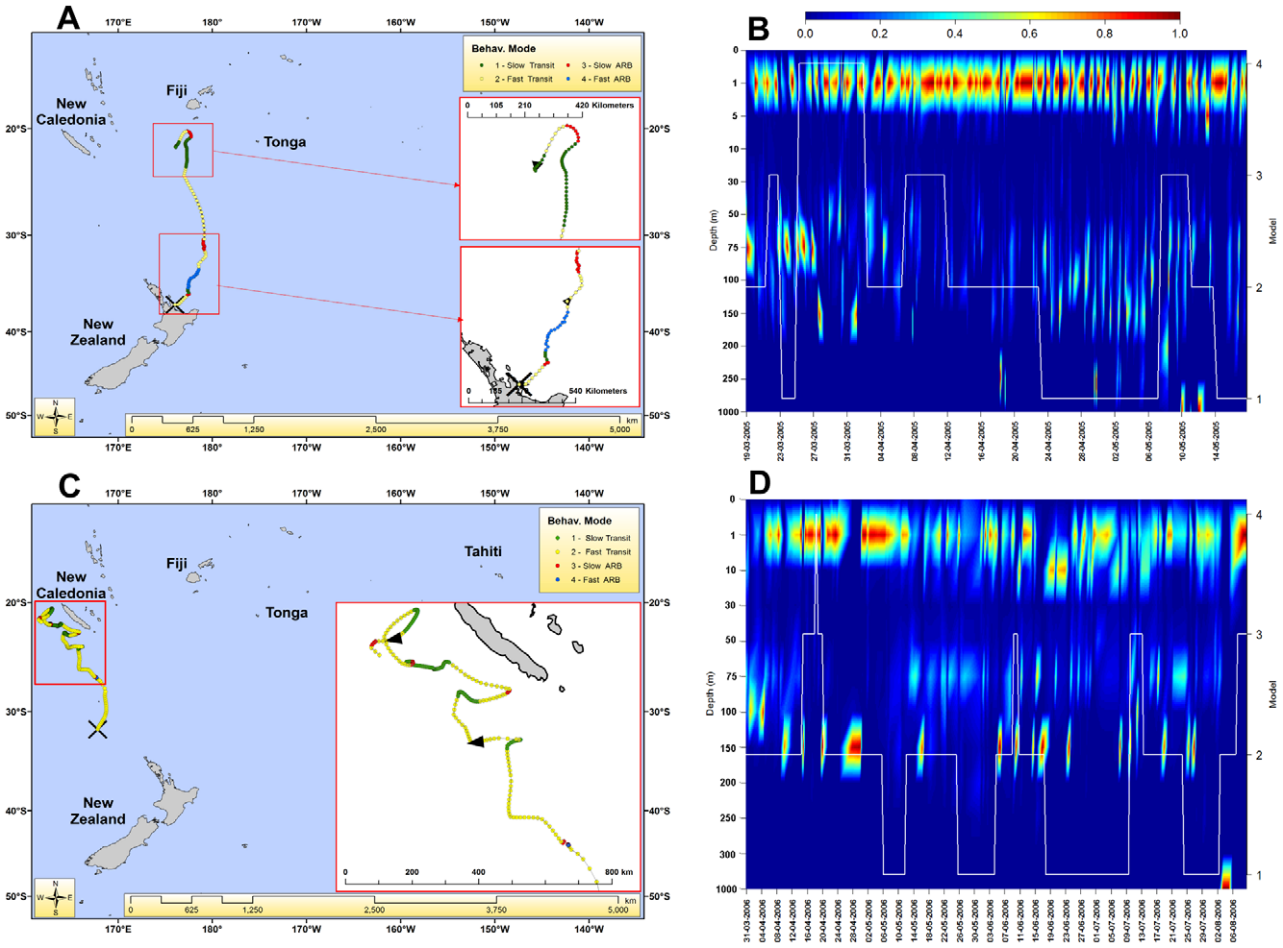
Individual striped marlin from 2005 and 2006 tagging seasons, representative of overall patterns. A. STM05.04 was tagged on 18 March 2005 and provided 60 days of data. Estimated behavioural modes are colour-coded and legend is at top-right. B. Time-at-depth profile for STM05.04. X-axis is time, primary Y-axis is depth (metres) of the water column, secondary Y-axis is estimated behavioural mode indicated with white trend-line, and colours are proportion of time (scaled 0–1, legend at top) spent at depth. C. STM06.10 was tagged on 31 March 2006 and provided 133 days of data. D. Time-at-depth profile for STM06.10.

STM05.4 was tagged in the western Bay of Plenty on 18 March 2005 (Figure 2A). It departed the tagging location in fast-transiting mode with ARB first observed 5 days after release and a total of 4 times before ending on 17 May 2005 approximately 500 km southwest of Fiji. During the first two weeks at liberty depth oscillated between the surface and *c.* 75–150 m. During the ensuing two weeks STM05.4 spent the majority of its time at or near the surface, with only brief forays below 50 m (Figure 2B). From 24 April 2005, its depths frequently oscillated between the surface and *c.* 75–150 m. The highest latitude reached was 20.2°S on 10 May 2005 which coincided with dives ≥250 m on 10 and 12 May before turning south and shifting from ARB to fast-transiting mode.

STM06.10 was tagged on 31 March 2006 in the Tasman Sea at the Wanganella Banks (Figure 2C). It spent the first 14 days post-release in fast-transiting mode before changing to ARB for 5 days in mid-April. ARB was inferred 4 separate times during the trajectory. STM06.10 spent the following four months moving north/northwest, switching between transiting and ARB as it moved west of New Caledonia until the track terminated on 11 August 2006. The highest latitude reached was 20.5°S on 25 July 2006, with dives ≥300 m on 4 August 2006, before turning south and changing from slow-transit mode to fast-transit mode. Swimming depth varied between the surface and 150 m throughout the trajectory (Figure 2D).

**Figure 3:**
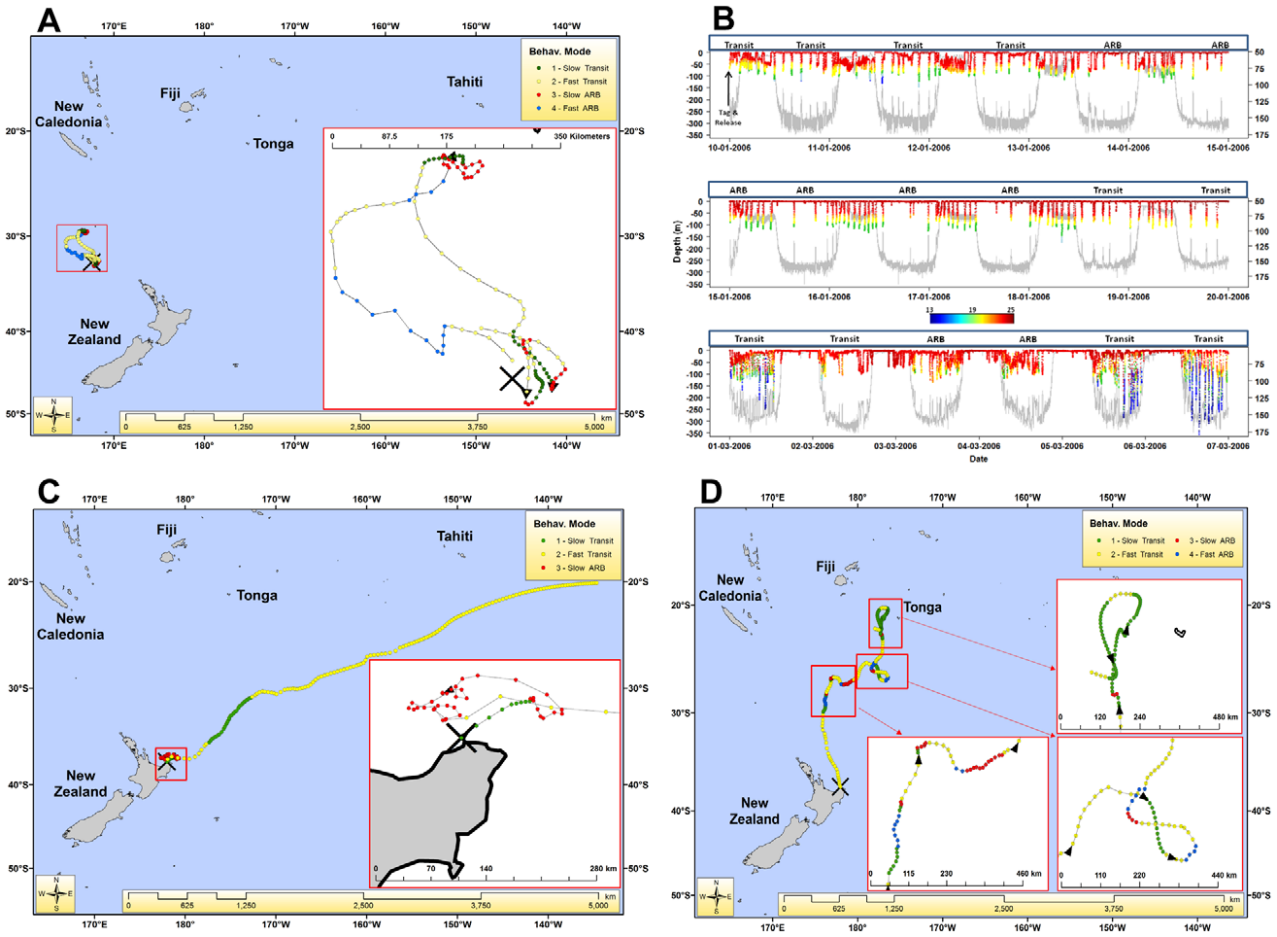
Representative individual striped marlin from 2006, 2007 and 2008 tagging seasons. A. STM06.01 was tagged on 10 January 2006 and provided 68 days of data. B. Archival time-series of temperature, depth and light-levels for STM06.01. Colored line represents water temperature with colors (legend in middle of plot) and depth (m) within the water column on the primary Y-axis; grey line shows light-level scaled in dimensionless units on the secondary Y-axis. C. STM07.01 was tagged on 19 February 2007 and provided 103 days of data. D. STM08.01 was tagged on 6 March 2008 and provided 115 days of data.

STM06.1 (Figure 3A) was double-tagged on 10 January 2006 in the Tasman Sea, and 25 days of SLRT and 68 days of PSAT data were recorded before mortality occurred on 18 March 2006. After death the marlin sank to 596 m depth and drifted deeper to 644 m over the following two days. The descent rate over 300 m was 3.3 meters/minute. Its PSAT began transmitting 97 km away from its tagging location and its archival data were recovered in early 2008, when the tag was discovered on a beach in eastern Australia. STM06.1 departed the tagging location in fast-transiting mode for the initial three days, followed by switching between fast-ARB and fast-transiting for 17 days before spending about 2 weeks circling a small area, eventually returning to very near its capture location. During the first 10 days after release its diving activity was irregular, with crepuscular patterns not well defined. Night diving frequencies were higher during this period than the rest of the record, while spending almost all of its time in the upper 100 m. From day 11 onwards its behavioural patterns became more characteristic of day/night activity. In the early part of the record, ARB was commonly associated with high dive frequencies (within upper 100 m) at night, and during daylight very close to the surface. Transiting behaviour was associated with regular diving throughout both day and night. Later in the record transiting was associated with many deeper and colder dives (up to 350 m) during the day, with ARB again correlated with high dive frequencies to shallow depths (≤100 m) during the day (Figure 3B).

STM07.1 was tagged on 19 February 2007 at Waihau Bay in the eastern Bay of Plenty (Figure 3C). Upon departing the capture location it moved slowly to the northeast for 5 days before entering slow-ARB mode and spending the following 18 days within 100 km of the New Zealand coast in the eastern Bay of Plenty. On 13 March 2007 it made a sudden directed movement away from the New Zealand coast, spending the following 2.5 months in transiting mode as it moved into the central Pacific Ocean. From mid May (*c.* 145°W, *c.* 22°S) its northerly progress slowed, with the final SLRT location observed on 2 June 2007.

STM08.1 was tagged on 6 March 2008 at Waihau Bay (Figure 3D). It departed its tagging location in fast transiting mode; ARB was first detected 16 days after release, and 7 times during the trajectory. From the beginning of April this fish began moving east/northeast, traveling in a figure-eight loop before continuing north through May. The highest latitude reached was 20.2°S on 12 June 2006 before changing from slow-transit to fast-transit mode while turning south, with the track terminating on 29 June 2006.

### Capture effects

**Figure 4:**
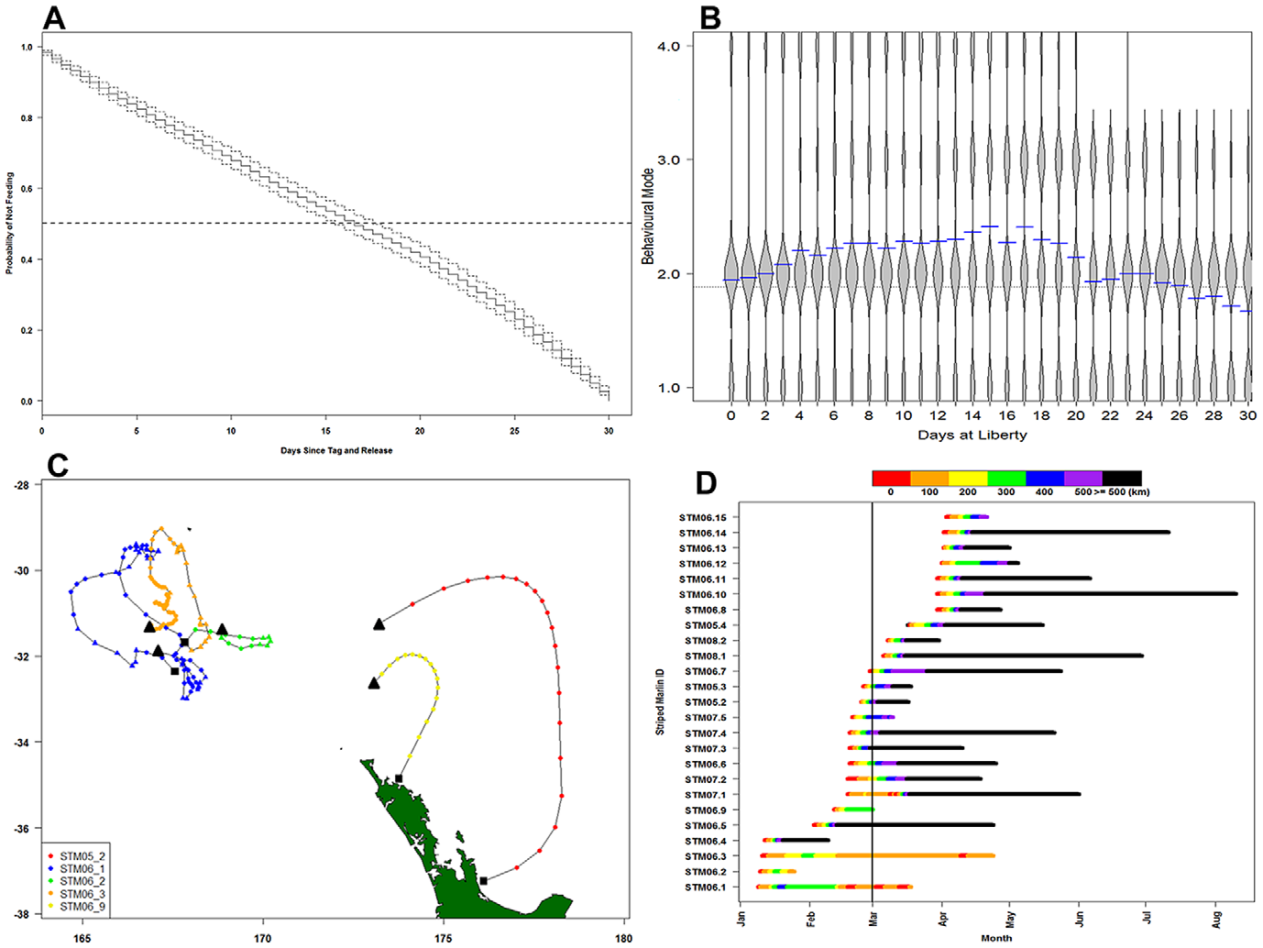
Capture effects on behaviour. A. Kaplan-Meier survival curve over first 30 days at liberty (solid stepped line) with 95% confidence intervals (dotted stepped lines). The probability of feeding through time is 1 - probability of not feeding (1 minus y-axis), with the odds of feeding or not estimated to be even (50∶50) at day 16 since release (straight horizontal dashed line). B. Proportions of striped marlin in behavioural modes 1–4 by days at liberty. For each day, width of bubbles (called ‘beans’) represents proportion of all pooled striped marlin in each behavioural mode and blue dashes are mean behavioural mode for all pooled marlin. C. Five striped marlin tagged before 1 March (2005 and 2006) which circled back towards their tagging locations. Black squares represent tagging locations and black triangles are final transmitted locations. D. Distance away from initial capture location for each marlin, ordered by calendar tagging date. Color blocks (legend at top) denote straight-line distance (km) from initial release location, and the vertical black line marks 1 March, denoting ‘early’ and ‘late’ season.

On average, a lower proportion of ARB was predicted during the first 16 days after release. Survival analysis indicated the probability of observing transiting and ARB behaviours was not equal (50∶50) until after 16 days post-release (Figure 4A). Proportions of fast-transiting behaviours were highest over a similar period, with proportions of slow-transiting and ARB behaviour increasing after approximately 16 days at liberty (Figure 4B). Travel speeds for tagged striped marlin during the initial 10 days post-release were significantly faster (1.97±1.20 km/h) than all subsequent periods (1.28±.93 km/h) (*p*≤0.001, θ = 11.8). Relative turning angles (angles between consecutive locations) were significantly lower during the first 10 days at liberty than all subsequent periods (*p* = 0.014, *t* = 2.45). Trajectories were aligned significantly more northwards during the first 10 days at liberty than all subsequent days (*p*≤0.001, t = 12.83). In addition to the strong tendency to move away from tagging locations, a seasonal trend was observed in the likelihood of marlin returning to their capture vicinity within the same tagging year. Of the marlin tagged before 1 March, 5/14 circled back to within 500 km of their capture location (Figure 4C). None tagged after 1 March returned to within 500 km of their capture locations during the same season (Figure 4D).

### Depth profiles

**Figure 5:**
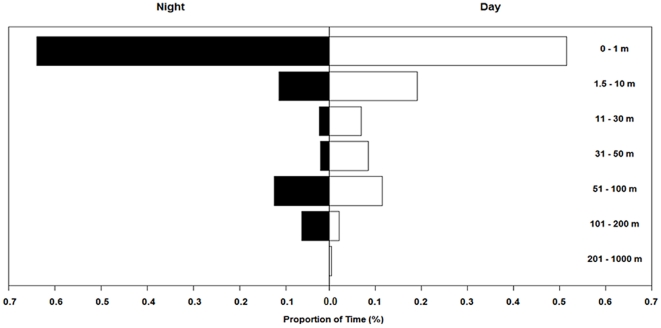
Proportion of time spent at depth (in meters) by day and night for all marlin tags reporting PSAT data between 2005 and 2008. Different tags were programmed to summarize data at 12 discrete depth intervals, and the seven bins shown here are aggregates of the bins that were common to all tags.

Maximum depths were significantly greater (*p*≤0.001) for transiting behaviours (127±57 m) than for ARB (108±49 m). Across all modes, they spent 51.4% of their time between within 1 m of the surface during the day, and 64.0% of their time in this range at night (Figure 5). However, they spent more time during night in the 50.5–200 m depth range (19.1%) than during the day (13.7%). The maximum depth observed overall was 460 m, where temperature at depth was 10.8°C, and surface temperature was 23.4°C.

### Northerly extent

**Figure 6:**
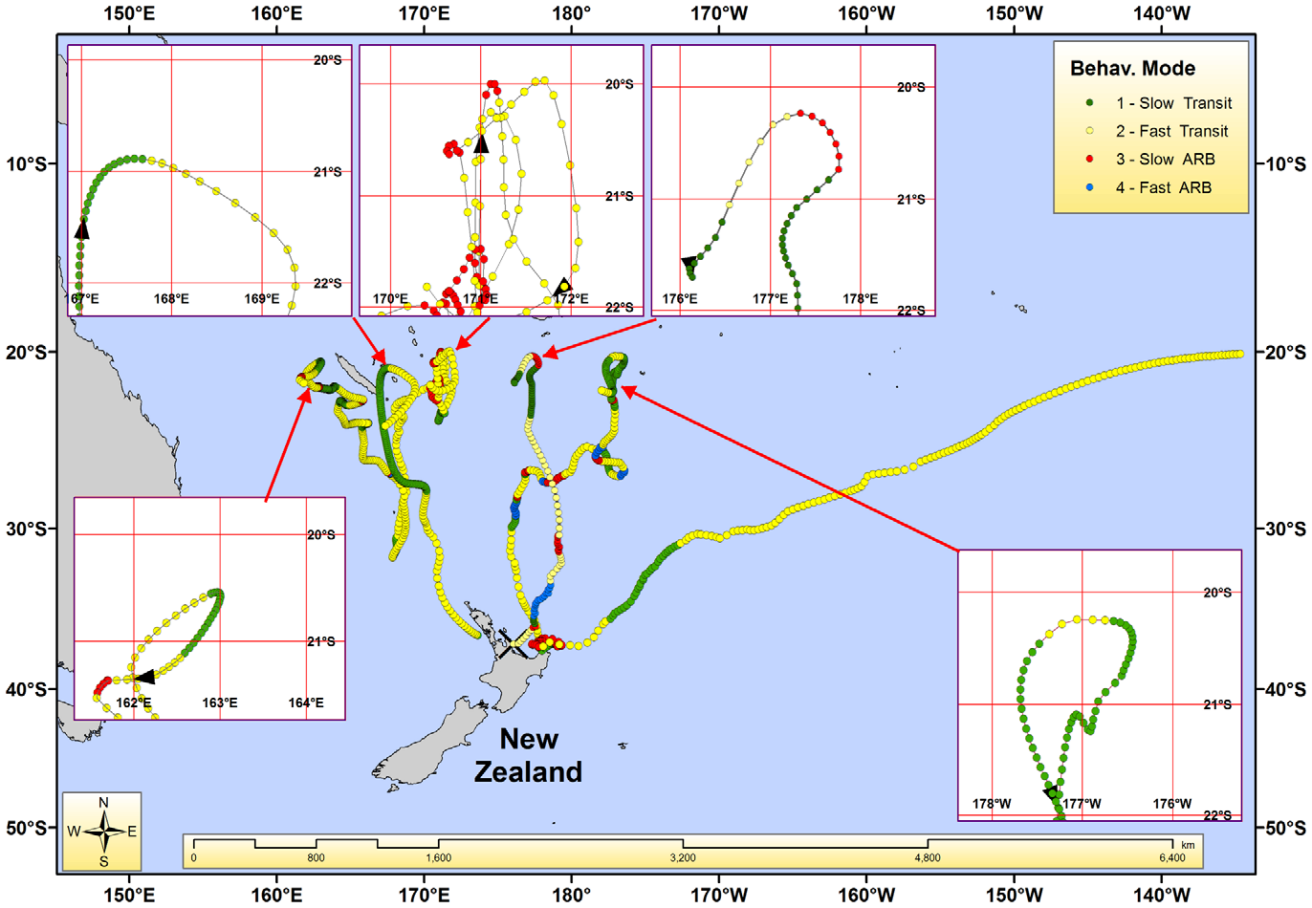
Six striped marlin trajectories (5 insets and one not inset) displaying either directional reversals or stopping northerly progress between 20–21°S latitude.

Five marlin (2005 = 1, 2006 = 3, 2008 = 1), initially tagged and captured in different months (February–April) and different locations (eastern NZ, Western NZ, Tasman Sea) moved towards the tropics and suddenly reversed directions at 20–21°S latitude upon arrival during the months April–August (Figure 6). These directional reversals coincided with a switch to fast-transiting mode in five fish. A sixth fish showed northerly progress leveling off at this latitude as the fish continued on a period 61 days of fast-transit (only trajectory not inset in Figure 6). A shift in depth distributions was apparent when PSAT data were available (n = 1, 2005; n = 3, 2006). At lower latitudes, depth plots show striped marlin spent most of their time at or near the surface (0–5 m). Approaching 20°S latitude movements tended toward subsurface depths of 5–10 m, or coincided with a deep spike dive below 250 m (Figures 2B and 2D).

## Discussion

### Data quality

Dealing with error-prone geolocations can be challenging, but Kalman filters enable the errors of marine telemetry techniques to be quantified and minimized [29], [33], [38]–[40] (see Appendix S1, Figures S1, S2 and S3, Tables S1 and S2). On average, CTCRW longitudes were 44 km closer to Argos longitudes than uKFSST longitudes were, with mean deviations from Argos of 12.5 km and 56.8 km respectively. Likewise, CTCRW latitudes were 24 km closer to Argos latitudes, than uKFSST latitudes were, on average (mean deviations were 87.8 km and 112.2 km respectively for uKFSST). The marked improvements in geolocation error distributions over those previously reported were due to geolocation processing at three levels. Firstly, calibrating approximations of light-level geolocations with Argos mid-points improved the initial estimates. With these initial estimates as inputs, uKFSST was then used to improve subsequent geolocation estimates, particularly with respect to latitude. Finally, the ability to parameterize the CTCRW model with error error terms for all location classes enabled further reduction of errors and estimation of trajectories on continuous time-scales. RMS longitude errors were reduced to near theoretical limits [26], [27], [41] and significantly below errors reported in other studies [2]–[4]. These studies did not calibrate light-level geolocations because their aim was to evaluate quality in the absence of calibration data. Furthermore, other studies have not used the third tier of location processing, state-space time-series regularization with algorithms such as the CTCRW [33]. The tendency of striped marlin to spend most of their time at the surface makes SLRT possible and maximizes the quality of light-level data for geolocation, however the effects of sea-state on the frequency and quality of SLRT are not well understood. However, the time-series of data available for behavioural modeling was significantly increased (doubled) by the inclusion of both Argos based SLRT and PSAT geolocation data.

STM06.1 made rapid and uncharacteristic west-to-east movements immediately upon release (Figure 3A). The magnitude of initial longitudinal movements (particularly in the initial 10 days) may have fallen outside the bounds normally expected by the light level geolocation algorithms used [26], [27], inducing a longitude estimation bias which could not be resolved by further refinement or uKFSST. However, archival latitude geolocation RMS errors for STM06.12 were substantially smaller, again by nearly a half an order of magnitude from raw uKFSST estimates. Some datasets were particularly challenging to analyze, and occasionally it is not possible to resolve these issues. However, double-tagging with SLRT and PSAT tags shows that these problems are uncommon, but should be acknowledged.

### Behavioural classification

In spite of the improved accuracy of our longitudes derived from light level geolocation, significant error remains for latitude estimates, which may lead to erroneous predictions of fast-ARB. Fast-ARB may not represent a real biological state, but rather a temporally autocorrelated series of location estimates of higher-than-average spatial error. Biologically relevant signals almost certainly lie within these segments, but the magnitude of measurement error precluded making biological inferences. Recently developed methods can reduce, or even eliminate bias in light level latitudes [39], [40], which will improve the ability of behavioural models to represent real biological patterns. Data of regular time-series are required by the segmentation algorithms used in this study, however interpolation of positions between observed locations introduces additional uncertainty and longer gaps between observations likely cause the model to over-estimate the proportion of transiting-mode behaviour.

ARB is representative of all behaviours such as foraging, resting and breeding that occur in spatially confined areas [42]. Data from the austral striped marlin breeding season [43], [44] were not available in this study, which excludes ARB being interpreted as breeding. Striped marlin are obligate ram ventilators [45] with high metabolic rates [46], thus are unlikely to remain in localized resting states for extended periods (days). A substantial proportion of ARB is likely to be foraging behavior, but this mode also probably includes activities such as searching patterns, social activity, and periods of reduced activity. Transiting states can represent searching patterns, migration, and flight from predators among other possibilities. The frequent correspondence of changes in inferred behavioural states with variations in depth is encouraging (Figures 2B and 2D) because this provides a measure of confidence that meaningful patterns are discernible. Correspondence of behavioural states with depth distributions has been observed in Atlantic leatherback turtles as well (*Dermochelys coriacea*) using switching state-space models (SSM) [47]. The ability to corroborate behavioural inferences derived from 2-dimensional models with data from a third dimension provides a measure of confidence in detection of behavioural state changes. Furthermore, this confidence provides impetus to incorporate as much information as possible in future models to generate more realistic behavioural estimates.

The segmentation model we used calculates the likelihood of a trajectory segment belonging to a specific behavioural mode, but does not estimate a posterior probability as do other approaches to classification [6], [47]. However, our model utilized more information (speed and turning angles, see Table S3 for movement parameter estimates), than indices such as first passage time [35] and straightness indices [48], and can be extended to incorporate other measures of behaviour including diving patterns. Schick *et al.* [49] considered the current status of modeling animal movement and where some hierarchical Bayesian modeling approaches might lead to further progress. Essentially, they envisioned a behavioural SSM with a resource selection function nested within the framework to govern switching probabilities. The approach used here, combined with resource selection analysis can provide the kind of a priori information to better inform an SSM as they envisaged.

### Foraging patterns

Transiting behaviour tended to correspond with changes from surface to deeper waters, while ARB mode generally was associated with shallower depths. Opportunities to feed can be sparsely distributed through space and time for pelagic predators, so foraging frequencies observed here probably reflect these conditions. Shoals of prey (*ie.* sardines and other planktivorous fish) may sometimes persist for days [50] and even weeks, which would promote occurrence of ARB. However, diurnal vertical migrations of prey such as squid may not lead to discernible patterns of ARB. Transiting behaviour classified by the model likely includes foraging periods characterized by different movement patterns. Stomach contents of striped marlin from New Zealand waters have revealed prey common to surface-to-mid water depths, benthic teleosts and squid [51]. Stomach-content analysis of North Pacific striped marlin showed surface dwelling prey are dominant components of the diet, but occurrence of prey from deeper reaches of the water column indicate periodic changes in foraging patterns [52]. Squid, sardines and mackerel have previously been identified as primary components of their diet in North American waters [53]–[55]. Difference among these prey types complicate detection of all foraging habits using behavioural models comprised on only two-dimensional location data.

Jonsen *et al.* [47] found that leatherback sea-turtles, when foraging, tended to reverse directions frequently. We did not often observe such behaviour in this study. Disparity in patterns of foraging between these two studies may reflect different strategies in how prey is obtained at the different trophic levels. An effect of these different strategies could be different spatio-temporal scales at which foraging occurs. As patchy prey are encountered, prey-capture behaviour might occur over smaller spatio-temporal scales than grazing. Predatory foraging might be reflected by lower degrees of trajectory autocorrelation than grazing. The outcome of these strategic trade-offs are that turtles feed on more abundant and accessible prey (*ie.* jellyfish) which have lower nutritive value, while marlin feed on less abundant but higher nutritive value targets like saury (Family: Scomberesocidae) and mackerels (Family: Scombridae). Accordingly, models designed to identify analogous behaviours (ie. foraging) which driven by different strategies (*ie.* predatory vs. grazing) probably require unique configurations.

Across all striped marlin, a mean of nearly 3 weeks separated periods of ARB activity. Although up to 50 days separated these periods it should not be assumed that this is truly reflective of foraging frequency given the range of prey types commonly pursued. From visceral warming in archival tagging data, Bestley *et al.* [56] noted southern bluefin tuna (*Thunnus maccoyii*) fed on 76% of days, with periods between confirmed feeding events ranging from 3.9–24.4 days (mean 11 days). The ability to detect feeding through such physiological data improves confidence in estimates of foraging success. The intervals of feeding success observed from endothermic animals such as tunas are probably quite reliable, and provide some indication of reasonable feeding/fasting intervals in other highly migratory pelagic fishes.

### Effects of capture and tagging

New Zealand's recreational catch rates of striped marlin peak around March and begin to diminish around April [57]. Japanese longline CPUE from the southwest Pacific Ocean shows relatively higher striped marlin abundance during summer/autumn and lower abundance during winter/spring around New Zealand [58]. Squire and Suzuki [21] proposed from these patterns that the population's movement trended towards southerly latitudes during summer/autumn. If these assemblages are motivated by foraging opportunities around New Zealand and the Tasman Sea, it could be expected that they would remain in these areas during these periods. A tendency of striped marlin to immediately depart capture locations in the southwest Pacific Ocean was initially detected by Sippel *et al.* [20] and multiple seasons of electronic tagging data show this to be a consistent pattern [4]. Tagged striped marlin tended to move north following release, while approximately 1/3 of those tagged during the summer circled back towards their capture locations, but none of those tagged in the autumn exhibited this behaviour. Similar patterns of departure in single tagged fish (PSAT only) where either documented or are apparent in [10], [20], indicating that double-tagging did not have a greater effect on movement patterns.

Movement out of range of domestic fishing vessels may also contribute to the particularly low recapture rates (0.52%) of striped marlin in the New Zealand cooperative gamefish tagging program [23]. Of the 28 tagged during 2005–2008, two mortalities (7%) occurred within 48 hours of capture and release. This confirms high post-release survivorship, and other factors such as tag shedding probably contribute more to low conventional-tag return rates. Three mortalities of the 25 surviving capture and tagging (12%) were observed more than 30 days after release (33, 53, 67 days). Transmitted temperature at depth records from two marlin showed temperatures of greater than 25°C, which remained constant at depths ranging from the surface to 600 m. These temperature and depth patterns are similar to an apparent predation of a PSAT tagged Opah (*Lampris guttatus*) by a warm bodied shark (*ie.* a lamnid shark) [59]. Predation is probably an important reason for non-reporting of PSAT tags in billfish telemetry studies.

Time-series analysis indicates that the probability of occurrence of ARB and transiting behaviours was not equal (50∶50) until 16 days after tagging. Bestley *et al.* [56] detected a 19±10 (mean ± SD) day period of post-release fasting (range 5–38 days). This is comparable to the 12.7 day period of diminished foraging probability detected from behavioural analysis of Atlantic leatherback sea turtle telemetry data [60], and are not unlike the 5–20 days of diminished body condition observed from short-term recaptures of conventionally tagged southern bluefin tuna [61]. Evidence for capture effects in other Istiophorid tagging data can be deduced from departures of white marlin (*Kajikia albidus*) [15], black marlin (*Makaira indica*) [62], and Pacific blue marlin (*Makaira nigricans*) [17] from their capture/tagging locations.

We hypothesize that capture and/or tagging triggered migration in at least half of striped marlin sooner than would be expected if they were not captured and tagged. Since it is not possible to know the long-term movements of individual untagged fish, the primary difficulty in assessing the effects of capture and tagging is the challenge of gathering behavioural control data. An approach to obtaining such data is free-tagging uncaptured fish (wild, freely swimming fish). If substantial differences in behaviour are observed between captured and uncaptured striped marlin, this would suggest that the capture process contributes to departures. Two striped marlin have been free-tagged with a PSAT by blue-water spear fishers to investigate movements of uncaptured fish. One provided short-term data which are suggestive of different movement patterns (see Appendix S2, Figure S4), and data were not received from the other tag. There are numerous important factors to consider when testing this hypothesis which include regional, temporal, and ontogenetic effects.

A comprehensive investigation into the effects of exhaustive exercise on post-release survivorship and blood-borne stress indicators on sharks, tunas and marlin found compelling evidence that recreationally caught gamefish species are physiologically compromised after release [63]. Wells *et al.* [64] measured elevated plasma electrolytes, blood glucose, lactate and haematocrit levels in moribund striped marlin sampled dockside at recreational fishing competitions. They provided useful measures of peak of stress indicators, but it is very difficult to get baseline measurements of these indicators in unstressed striped marlin.

Because the effects of capture and tagging on animals behaviour and fitness are not well understood, further investigation is merited. Furthermore, capture effects that trigger early departure from the tagging region could bias spatio-temporal data used in management and stock assessment.

### Depth and catchability

Utilization of the water column by striped marlin is mediated heavily by two primary oceanographic conditions, oxygen saturation and temperature relative to the surface. The maximum depth reported from PSAT tagging in the ETP was 192 m, with only 9% (4/45) of individuals reported to exceed 150 m depth [14], while no descents below 150 m were observed from acoustic tracking there and in the central Pacific [11], [12]. Shallow oxyclines and thermoclines are uncommon in the southwest Pacific [65], and consequently our data show all striped marlin exceeded 150 m depth, descents to deeper than 200 m were common, and the maximum depth observed was 460 m. Like elsewhere, these fish spent more than half of their time within 10 m of the surface, but a second smaller peak (15%) was observed between 50–100 m (Figure 6) which has not been previously observed. Geographic variation of depth distribution has important consequences.

Among the uncertainties of the first southwest-Pacific stock assessment was vertical distribution which effects vulnerability to fishing activity [66]. Data cited about utilization of the water column was sourced from telemetry data in the ETP where striped marlin spend most of their time in the upper 40 m. We show occupancy of the water column differs substantially in the southwest Pacific, which should alter assumptions about susceptibility to fishing activity in stock-assessment. Regionally specific telemetry data can be useful for understanding gear selectivity and standardizing CPUE for billfish stock-assessments [67]. For example, relative CPUE for striped marlin from the Hawaii longline fishery was quite small at depths ≥120 m compared to shallower depths [68], but our data indicate these trends would probably be different in the southwest Pacific. Regionally standardized CPUE might be an improvement for future stock-assessments. Furthermore, it has been estimated that commercial bycatch accounts for around 90% of adult marlin mortality, and attempts to mitigate this will be affected by geographically variable vulnerability to gear at depth [69].

Gear depth is a consideration in recreational catch rates as well. Recreational billfishers usually troll for billfish at the surface, but we show how fishing activity targeted there would probably fail to attract the attention of marlin when they move deeper. This is a likely explanation for the marlin bite turning on and off, and innovations to recreational fishing techniques and gear might provide new opportunities to increase catches at depth.

### Northerly extent

A 10–20° latitudinal band north and south of the equator is commonly recognized as a break in the geographic distribution of adult striped marlin in the central and western Pacific [21]. It is striking that a pattern of direction reversal or apparent termination of northerly progress between 20–21°S latitude was repeatedly observed in each season (2005–2008). Changes to fast-transiting mode coinciding with directional reversals suggest an individual response. Striped marlin moving further north than 20°S latitude have previously been documented [20], primarily during spring [10]. We did not acquire data during the spring, but oceanographic conditions probably help explain this pattern. Any number of combined oceanographic variables (*ie.* increased mixed layer depth and temperature, decline in oxygen saturation, changing upwelling/downwelling conditions, etc.) might cue these behaviour changes. The probable influence of oceanography on population structure and stratification demonstrates how environmental factors can be important inputs in population dynamics models [70]. The discontinuity in adult distribution observed in the southwest Pacific ocean is worth considering for spatial stratification of future stock-assessments.

## Supporting Information

Figure S1 **Joint probability densities of errors for CTCRW regularized uKFSST geolocations from transmitted PSAT data.**
(TIF)

Figure S2 **Effects of CTCRW regularization on double-tagged striped marlin STM06.14.** The black line and points are raw location data, with triangles representing uKFSST location estimates and circles representing Argos locations from the SLRT tag. Red represents the smoothed CTCRW pathway.
(TIF)

Figure S3 **Striped marlin trajectories by season 2005–2008 after CTCRW regularization.**
(TIF)

Figure S4 **Controlling for striped marlin capture effects.** Red vector (red inset box): Travel distance and direction over 8 day PSAT deployment from speargun. Black vectors: Travel distance and direction over initial 8 days at liberty for six individuals captured with standard recreational fishing methods.
(TIFF)

Table S1 **Multiplier function K values used as error model parameters used in CTCRW regularization.**
(XLS)

Table S2 **Estimates of t-distribution parameters from non-linear minimization for uKFSST longitude and latitude errors.**
(XLS)

Table S3 **Density function parameters estimated by maximum likelihood for each individual.**
(XLS)

Appendix S1 **Temporal regularization and behavioural classification of striped marlin trajectories.**
(DOC)

Appendix S2 **Comparison of free-tagged to recreationally captured and tagged striped marlin movements.**
(DOC)
