# Supplementary figures and images for: Investigating Behaviour and Population Dynamics of Striped Marlin (Kajikia audax) from the Southwest Pacific Ocean with Satellite Tags
Source: PLoS One. 2011 Jun 14;6(6):e21087. doi: 10.1371/journal.pone.0021087 (PMC3114854; doi:10.1371/journal.pone.0021087)

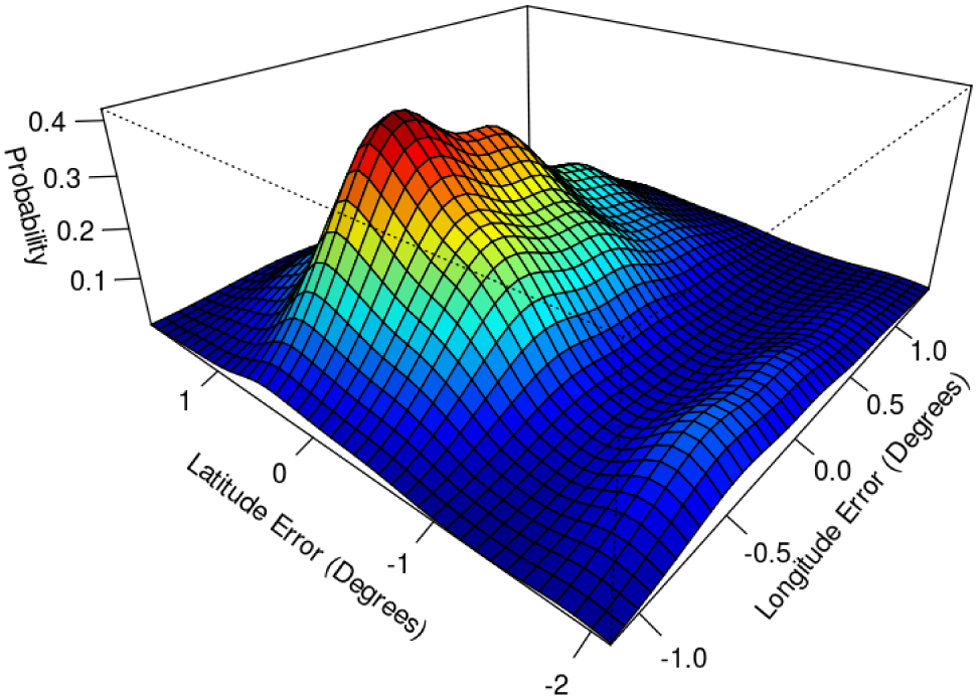

Supplement: Figure S1 — Joint probability densities of errors for CTCRW regularized uKFSST geolocations from transmitted PSAT data. (TIF) [file pone.0021087.s001.tif]

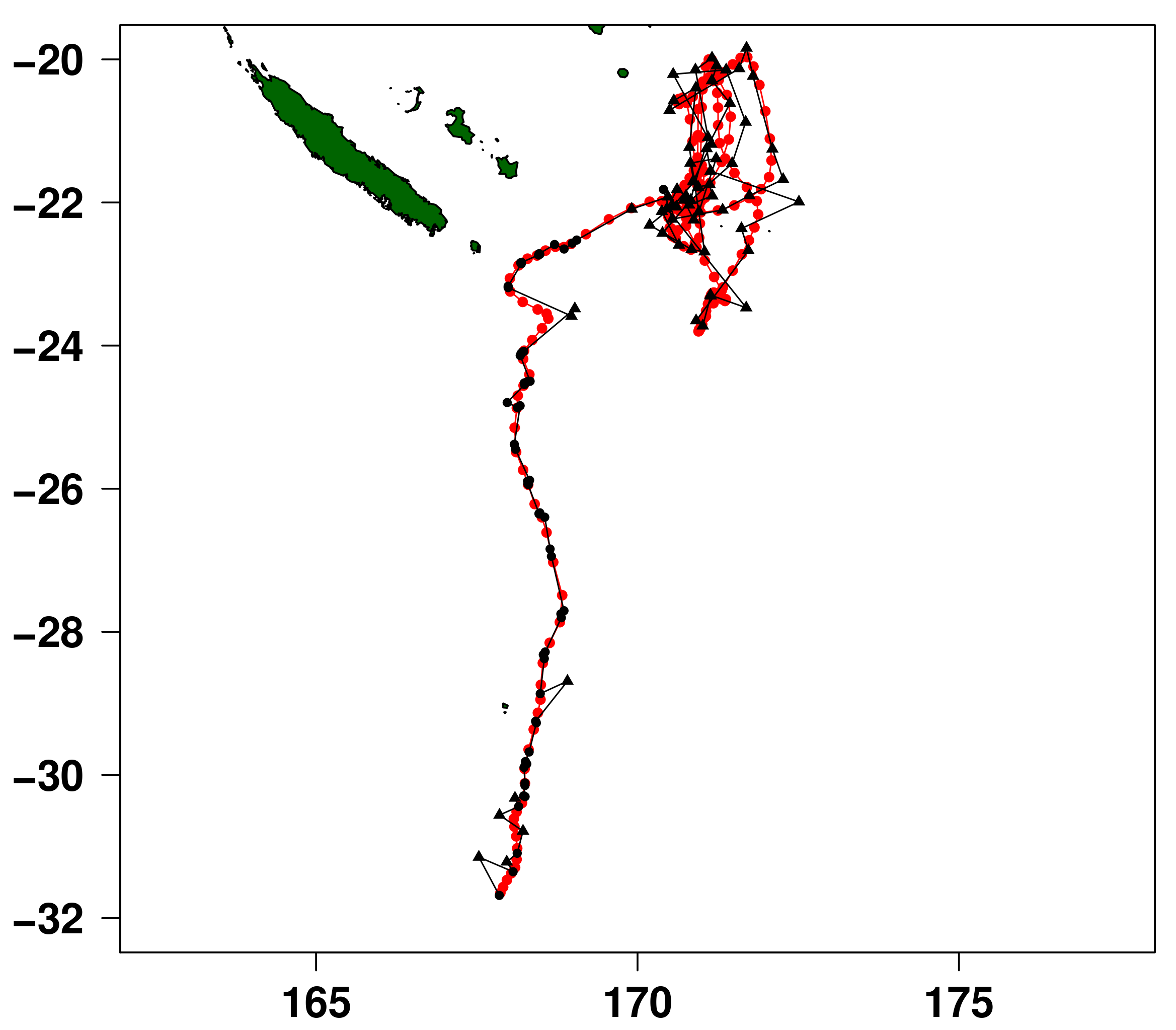

Supplement: Figure S2 — Effects of CTCRW regularization on double-tagged striped marlin STM06.14. The black line and points are raw location data, with triangles representing uKFSST location estimates and circles representing Argos locations from the SLRT tag. Red represents the smoothed CTCRW pathway. (TIF) [file pone.0021087.s002.tif]

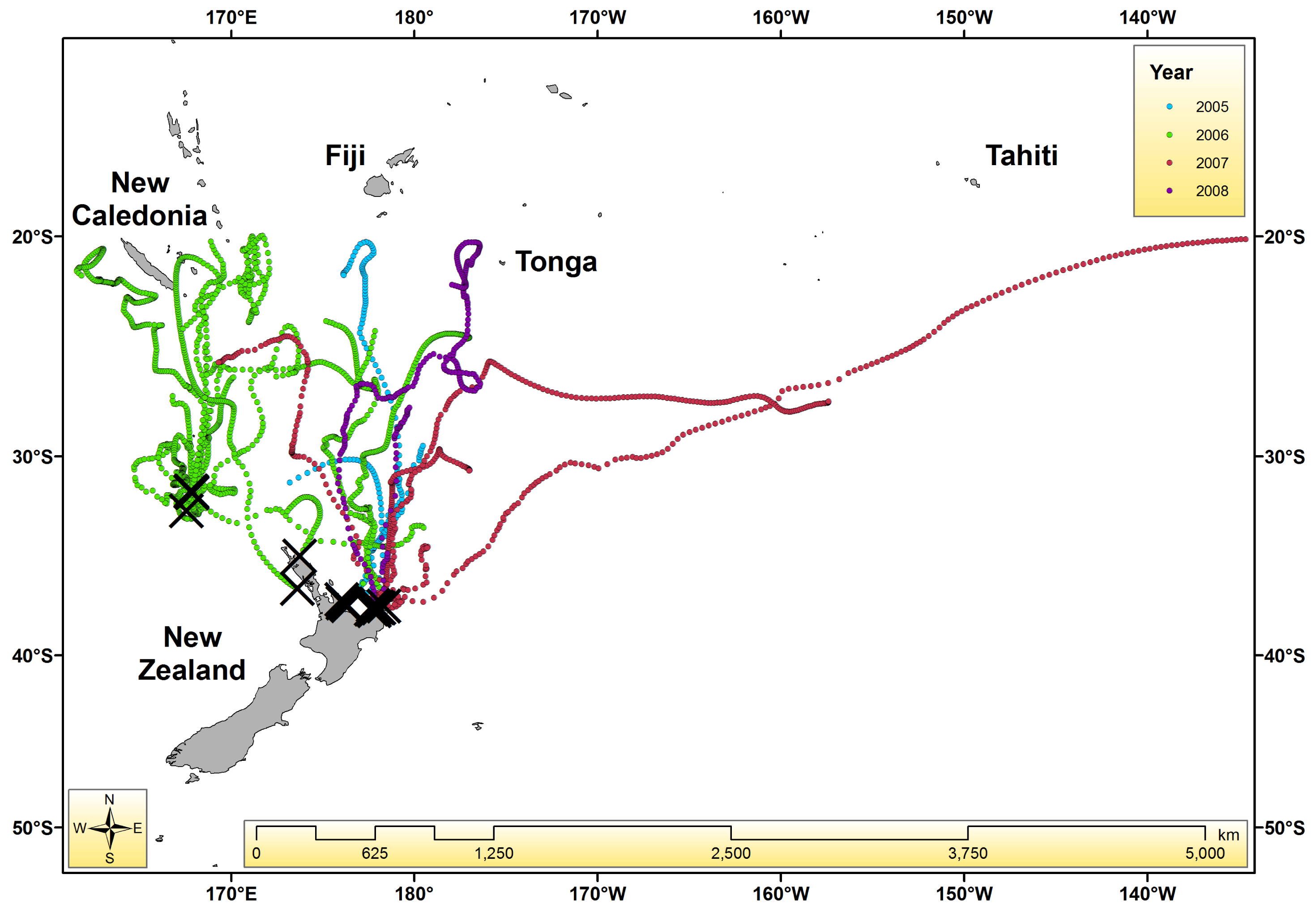

Supplement: Figure S3 — Striped marlin trajectories by season 2005–2008 after CTCRW regularization. (TIF) [file pone.0021087.s003.tif]

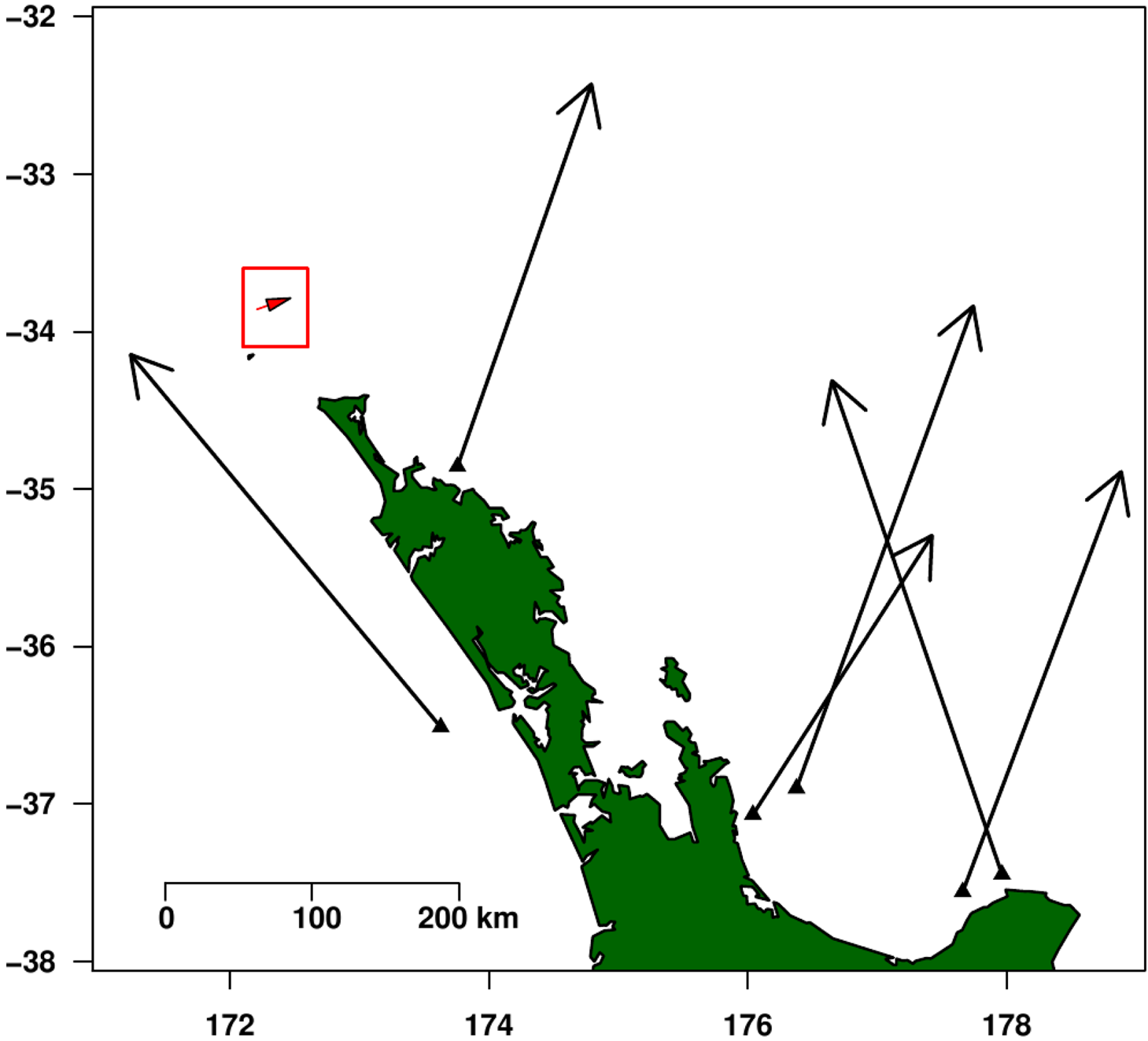

Supplement: Figure S4 — Controlling for striped marlin capture effects. Red vector (red inset box): Travel distance and direction over 8 day PSAT deployment from speargun. Black vectors: Travel distance and direction over initial 8 days at liberty for six individuals captured with standard recreational fishing methods. (TIFF) [file pone.0021087.s004.tiff]
