# Supplementary material for: Investigating Behaviour and Population Dynamics of Striped Marlin (Kajikia audax) from the Southwest Pacific Ocean with Satellite Tags
Source: PLoS One. 2011 Jun 14;6(6):e21087. doi: 10.1371/journal.pone.0021087 (PMC3114854; doi:10.1371/journal.pone.0021087)
Supplement: Appendix S1 — Temporal regularization and behavioural classification of striped marlin trajectories. (DOC) [file pone.0021087.s008.doc]

**Investigating Behaviour and Population Dynamics of Striped Marlin (*Kajikia audax*) from the Southwest Pacific Ocean with Satellite Tags**

Tim Sippel, John Holdsworth, Todd Dennis, John Montgomery

## Supplement A

### Data regularization

Prior to regularization, trajectories were filtered for outliers using the function sda(**argosfilter**) [1,2]. Outlier rejection criteria included Argos LC Z, speeds faster than 2 m/s, and angle spikes of 15 ° and 25 ° associated with spike distances of 2500 m and 5000 m.

The CTCRW regularization model was specified by three components: a movement model, a drift model (with animal velocity and drift velocity components), and a location error model (with longitude and latitude components). The movement model specified how movement parameters should vary through time as a function of time dependent covariates (the error and drift models). The drift model allowed for random deviations due to oceanic currents to effect the movement process, and animal velocity and drift parameters were initialized at zero. The error model was parameterized with standard deviation constants (K) associated with different location quality indices (Argos, uKFSST, GPS). The same K values given in the documentation for the function crwMLE(**crawl**) [3] were used for Argos positions (LC 3, 2, 1, 0, A, B). K for uKFSST geolocations were chosen by testing the constants 30, 40, 50, 60, 70, 80, 90, 100, and selecting the values which produced the smallest mean deviation from concurrent Argos positions (available within 24 hours, excluding LC Z). All error model parameters for longitude and latitude are found in Table S1, and the joint density distribution of uKFSST longitude and latitude errors is shown in Figure S1. Non-linear minimisation was also used to estimate t-distribution parameters for uKFSST longitude and latitude geolocation errors when SLRT derived Argos locations were concurrently available within 24 hours (Table S2). The before and after effects of CTCRW regularization on the double-tagging dataset for marlin STM06.14 are illustrated in Figure S2, and all smoothed trajectories shown in Figure S3.

### Behaviour classification

To classify trajectory segments into discrete pseudo-behaviours, Bayesian partitioning algorithms implemented in **adehabitat** [4,5] were used. This segmentation approach was developed for partitioning DNA sequences into homogeneous segments (ie. genes) [6]. In the context of trajectory classification, the process entailed simulating correlated random-walks (CRW) parameterized by probability distributions of the observed pathway characteristics (speed and turning angle), and using the segmentation algorithms to maximize the likelihood that any given segment of an observed trajectory belongs to one of these a priori models.

We were interested in distinguishing between phases of relatively 'slow', 'fast', 'straight' and 'not straight' movement phases. Animal movement theory indicates behaviours like migration, dispersal, or fleeing tend to be characterized by relatively straight movement pathways [7], and tortuous paths are representative of behaviours like resting, foraging and breeding [8]. Changes in speed are also useful metrics of animal behaviour. If relatively low and high magnitudes of both turning and speed are discretized, an array of four possibilities is created: 'slow-straight', 'slow-turning', 'fast-straight', and 'fast-turning', which were given more biologically relevant terms; 'slow-transit', 'slow-ARB', 'fast-transit', and 'fast-ARB' respectively. Others have referred to trajectory segments with high turning angles as 'area-restricted search' (ARS) [9,10]. The term 'search' is suggestive of a specific kind of behavior (searching), but the animal may or may not have found what it 'searches' for at any moment and classifying resting as a form of 'search' seems undesirable. Due to these semantics, the term 'area-restricted behavior' (ARB) is used as a more generic reference to area-restricted patterns.

The Weibull probability distribution
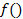
 has been used to model animal speed,
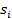
, in previous behavioural classification algorithms [11], and it fit these data also. Weibull scale,
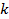
, and shape ,
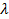
, parameters were estimated by maximum likelihood using the function fitdistr(**MASS**) [12], with ’slow’ and ’fast’ segregated at the median (Table S3). A wrapped Cauchy distribution
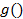
, was used to model turning angles,
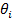
, segregating 'straight' and 'not straight' at the mean. Wrapped Cauchy concentration parameters,
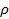
, for straight and turning were fixed at 0.97 and 0.03, with mean turning angles,
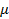
, centered at 0 ° and 180 °, respectively. Following this, the joint probability density function,
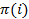
, of speed and turning angle is:


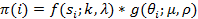


After setting up these hypothetical movement models, three functions were used to segment a telemetry pathway. The function modpartltraj(**adehabitat**) generated CRW movement models, drawing step lengths and turning angles from the prescribed distributions. Next, the number of trajectory segments were estimated by the function bestpartmod(**adehabitat**), which produced a log-likelihood for a range of possible segment counts in the trajectory. The number of segments for which the likelihood was maximized was considered to be the best estimate. Because the segmentation algorithm tended to overestimate the number of segments [6], Monte Carlo randomization as incorporated into the function to compensate for this bias. Since the correction tended to produce variable results when iterated multiple times, it was repeated 10 times for each trajectory using 1000 MCMC samples and the most conservative number of segments was considered optimal. Using this estimated number of switches, the function partmod.ltraj(**adehabitat**) predicted where discrete phases occurred within the trajectory.

**Literature Cited**

1. Freitas C (2010) argosfilter: Argos locations filter.. Available: http://CRAN.R-project.org/.

2. Freitas C, Lydersen C, Fedak MA, Kovacs KM (2008) A simple new algorithm to filter marine mammal Argos locations. Marine Mammal Science 24: 315-325.

3. Johnson, D. (2008) crawl: Fit continuous-time correlated random walk models for animal movement data.. Available: R package version 1.0-1.

4. Calenge C (2007) Exploring habitat selection by wildlife with adehabitat. Journal of Statistical Software 22: 2-19.

5. Calenge C (2006) The package “adehabitat” for the R software: A tool for the analysis of space and habitat use by animals. Ecological Modelling 197: 516-519.

6. Gueguen L (2001) Segmentation by maximal predictive partitioning according to composition biases. Computational Biology 2066/2001: 32-44.

7. Doerr ED, Doerr VAJ (2005) Dispersal range analysis: quantifying individual variation in dispersal behaviour. Oecologia 142: 1–10.

8. Ward D, Saltz D (1994) Forging at Different Spatial Scales: Dorcas Gazelles Foraging for Lilies in the Negev Desert. Ecology 75: 48–58.

9. Fauchald P, Tveraa T (2003) Using first-passage time in the analysis of area-restricted search and habitat selection. Ecology 84: 282-288.

10. Kareiva P, Odell G (1987) Swarms of predators exhibit“ preytaxis” if individual predators use area-restricted search. The American Naturalist 130: 233–270.

11. Morales JM, Haydon DT, Frair J, Holsinger KE, Fryxell JM (2004) Extracting more out of relocation data: building movement models as mixtures of random walks. Ecology 85: 2436-2445.

12. Venables WN, Ripley BD (2002) Modern Applied Statistics with S. Springer. p. Available: <http://www.stats.ox.ac.uk/pub/MASS4>.

### Behaviour classification model

# Author: Tim Sippel (tsippel@gmail.com)

# This R code (http://www.r-project.org/) classifies a trajectory at regular time intervals into

# four modes, based on changes in speed and relative turning angles. Alternative assumptions

# about parameter distributions can be used by substituting different density functions.

library(adehabitat)

library(MASS)

library(CircStats)

# The following code assumes animal trajectories are provided at regular time intervals.

# Assumes an object called 'data' has been created with the following four columns:

# x (longitude), y (latitude), date (format 'YYYY-MM-DD HH:MM:SS'), id (animal id).

data=as.ltraj(xy=as.data.frame(cbind(data$x, data$y)), date=as.POSIXct(data$date, 'GMT'),

id=data$id, burst=data$id, typeII=T, slsp="remove")

# Set distributions of angle means, centred at 0 degrees for 'transiting' and 180 degrees

# for 'ARB'

angle.means<-c(rad(0), rad(0), rad(180), rad(180))

# Estimate shape and scale parameters for Weibull distributions of step lengths

# (same as speed at regular intervals). 'slow' and 'fast' movements are broken by the

# median step length.

slow.shape=fitdistr(na.omit(data[[1]]$dist[data[[1]]$dist <

median(data[[1]]$dist, na.rm=T)]), densfun='weibull')$estimate[1]

slow.shape.sd=fitdistr(na.omit(data[[1]]$dist[data[[1]]$dist <

median(data[[1]]$dist, na.rm=T)]), densfun='weibull')$sd[1]

fast.shape=fitdistr(na.omit(data[[1]]$dist[data[[1]]$dist >

median(data[[1]]$dist, na.rm=T)]), densfun='weibull')$estimate[1]

fast.shape.sd=fitdistr(na.omit(data[[1]]$dist[data[[1]]$dist >

median(data[[1]]$dist, na.rm=T)]), densfun='weibull')$sd[1]

slow.scale=fitdistr(na.omit(data[[1]]$dist[data[[1]]$dist <

median(data[[1]]$dist, na.rm=T)]), densfun='weibull')$estimate[2]

slow.scale.sd=fitdistr(na.omit(data[[1]]$dist[data[[1]]$dist <

median(data[[1]]$dist, na.rm=T)]), densfun='weibull')$sd[2]

fast.scale=fitdistr(na.omit(data[[1]]$dist[data[[1]]$dist >

median(data[[1]]$dist, na.rm=T)]), densfun='weibull')$estimate[2]

fast.scale.sd=fitdistr(na.omit(data[[1]]$dist[data[[1]]$dist >

median(data[[1]]$dist, na.rm=T)]), densfun='weibull')$sd[2]

# Set concentration parameters for wrapped Cauchy distributions of turning angles.

straight=0.97

turn=0.03

# Define the movement model for 4 different modes, called 'slow-transiting, fast-transiting,

# slow-ARB, fast-ARB' in the publication.

move.mod=as.list(paste("dweibull(x=dist, shape="

,rep(c(slow.shape, fast.shape), 2)

,", scale="

,rep(c(slow.scale, fast.scale), 2)

,") * dwrpcauchy(theta=rel.angle, mu="

,angle.means,", rho=",c(rep(straight, 2), rep(turn,2)),")"))

# Use telemetry data and pre-defined movement model to initialize correlated random-walk.

mods.joint=modpartltraj(data, limod = move.mod)

# Estimate the 'optimum' number of segments, based on the assumed movement model.

part.joint=bestpartmod(mods.joint, Km = 40, plotit = T, correction = T, nrep = 2000)

# Estimate the extent of each segment within the trajectory.

traj.joint=partmod.ltraj(data, 20, mods.joint, 'locf')

# extract behaviour estimates (obtain package 'zoo' by typing install.packages('zoo'))

library(zoo)

model=as.vector(rep(NA, times=nrow(data.ltraj[[1]])))

model[traj.joint$stats$locs]=traj.joint$stats$mod

model=na.locf(model)

unloadNamespace('zoo')

# need to unload 'zoo' package here due to conflict with 'adehabitat' package

# create object to contain model outputs

data.out=as.data.frame(cbind(data[[1]],year=substr(data[[1]]$date, 1, 4),

month=substr(data[[1]]$date, 6, 7), day=substr(data[[1]]$date, 9, 10),

hour=substr(data[[1]]$date, 12, 13), minute=substr(data[[1]]$date, 15, 16),

second=substr(data[[1]]$date, 18, 19),model))

# Define colours for plot of behavioural modes.

data.out$col[data.out$model == '1']='green'

data.out$col[data.out$model == '2']='yellow2'

data.out$col[data.out$model == '3']='red'

data.out$col[data.out$model == '4']='blue'

# Plot behaviour estimates

plot(x=data.out$x, y=data.out$y, xlab="Longitude", ylab="Latitude",

main=paste(data.traj$id), type='l', las=1, font.lab=2)

points(x=data.out$x, y=data.out$y, col=data.out$col, cex=1.0, pch=16)

points(x=c(data.out$x[1], data.out$x[length(data.out$x)]), y=c(data.out$y[1],

data.out$y[length(data.out$y)]), pch=c(17,15), cex=1.4)

legend('topright', legend=c('Start', 'Slow-Trans', 'Fast-Trans', 'Slow-ARB', 'Fast-ARB', 'End'),

pch=c(17,16,16,16,16,15), cex=1.1, col=c('black', 'green', 'yellow2', 'red', 'blue', 'black'))
