# Supplementary material for: Investigating Behaviour and Population Dynamics of Striped Marlin (Kajikia audax) from the Southwest Pacific Ocean with Satellite Tags
Source: PLoS One. 2011 Jun 14;6(6):e21087. doi: 10.1371/journal.pone.0021087 (PMC3114854; doi:10.1371/journal.pone.0021087)
Supplement: Appendix S2 — Comparison of free-tagged to recreationally captured and tagged striped marlin movements. (DOC) [file pone.0021087.s009.doc]

**Investigating Behaviour and Population Dynamics of Striped Marlin (*Kajikia audax*) from the Southwest Pacific Ocean with Satellite Tags**

Tim Sippel, John Holdsworth, Todd Dennis, John Montgomery

### Capture effects

A speargun was used to PSAT tag a striped marlin on 15 February 2010 off of the Three Kings Islands, without capture on fishing gear. The tag transmitted after 8 days at liberty (premature to its intended 30 deployment), 16.6 km northeast (heading = 71°) of its tagging location (Figure S4.). Upon premature detachment the tag drifted at the surface for 48 hours before transmitting, which would minimally alter the calculated distance, but could change the heading significantly. For comparison, six individuals from this study (2005, *n*=1; 2006, *n*=3; 2007, *n*=1; 2008, *n*=1) traveled on average 317 ± 51 km from their capture location over the first 8 days at liberty on a average heading of 5.7 ± 28.6 degrees.

## Acknowledgements

Thanks to Mike Gattanella of Hoff Productions, Ltd (USA) and his crew for making it happen. Thanks to Dean Martin and Steve Hathaway for tagging the fish.
